# Bilateral Congenital Diaphragmatic Hernia

**Published:** 2012-09-01

**Authors:** Anjan kumar Dhua, Satish K Aggarwal, NB Mathur, GR Sethi

**Affiliations:** Department of Pediatric surgery, Maulana Azad Medical College, Delhi 110002.; Department of Pediatric surgery, Maulana Azad Medical College, Delhi 110002.; Department of Pediatrics, Maulana Azad Medical College, Delhi 110002.; Department of Pediatrics, Maulana Azad Medical College, Delhi 110002.

**Keywords:** Bilateral congenital diaphragmatic hernia, Pulmonary hypertension, Silo

## Abstract

Bilateral congenital diaphragmatic hernia (CDH) is a rare birth defect, with a poor prognosis. We describe a case of bilateral CDH discovered while repairing the right sided CDH. Diaphragmatic defect was repaired and a silo was applied on the abdominal wound to avoid abdominal compartment syndrome. The patient however died postoperatively due to severe pulmonary hypertension.

## INTRODUCTION

Congenital diaphragmatic hernia occurs in every 2000-3000 live births [1]. Bilateral CDH is extremely rare, constituting less than 1% of all CDH cases [2]. Most of them die in utero while less than 35% survive [3,4]. The management of bilateral CDH is a challenge for pediatric surgeons and the neonatologists. We report a case of bilateral CDH with sac on both sides. 

## CASE REPORT

A male baby was born to a 30-year-old second gravida by normal vaginal delivery, in a private hospital. The baby had birth asphyxia and resuscitated with bag and mask ventilation. APGAR scores were 6 and 7 at 3 and 5 minutes, respectively. Patient was kept in the intensive care unit under oxygen hood and feeds were started.

He was referred to our hospital on day 6 with persistent respiratory distress. On admission his respiratory rate was 70/min, SpO2 was 87% with oxygen hood. The abdomen was soft and not distended. Air entry on both sides was reduced, more so on the right side. Liver dullness was noted abnormally high in the chest. A chest radiograph suggested right sided diaphragmatic eventration. Patient was intubated and ventilation started. A repeat radiograph showed elevated right hemi-diaphragm and shift of mediastinum to the left (Fig. 1A). Echocardiography revealed no cardiac abnormality.

**Figure:**
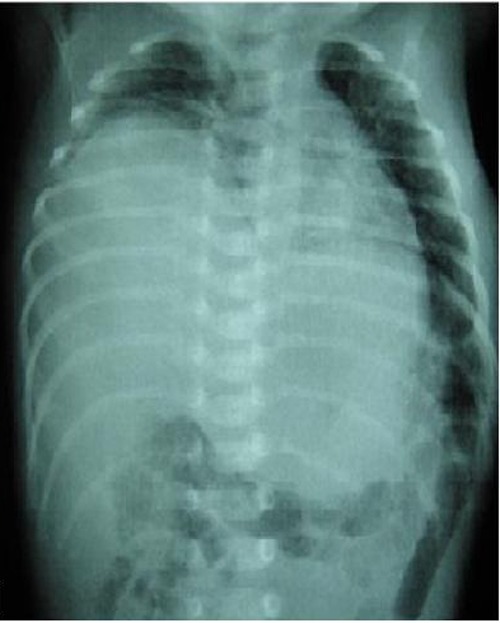
Figure 1: Preoperative chest radiograph.

After preoperative stabilization the baby was operat-ed through a right sub-costal incision. The liver was very large and difficult to manipulate, hence the incision was extended in a roof top fashion. Falciform ligament was divided to mobilize the liver. It was discovered that a large part of right lobe of liver was inside the right hemithorax. The intrathoracic portion of right liver was reduced into the abdomen by gentle manipulation. This revealed a well defined defect in the diaphragm with well formed anterior and posterior lips of the muscle of the diaphragm. The lung was visible through a transparent sac (Fig. 2A). Manipulation of the left lobe of liver revealed a diaphragmatic hernia on the left side as well. The content of left hernia was part of left lobe of liver, spleen and a small part of splenic flexure of colon. There was a sac covering the contents. 

**Figure:**
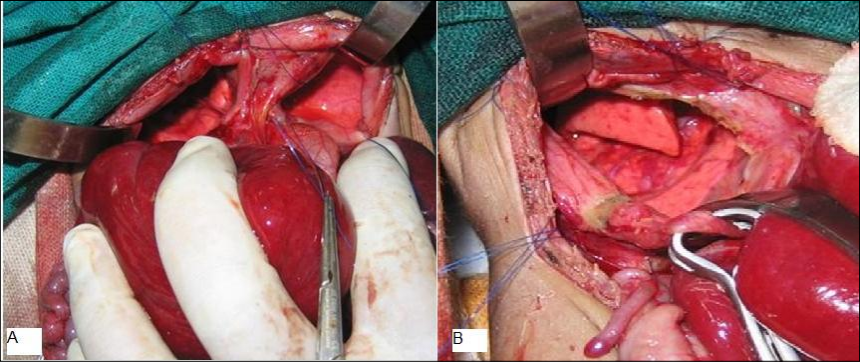
Figure 2: (A) Bilateral CDH with sac. (B) After excision of sac fairly well developed lung seen.

**Figure:**
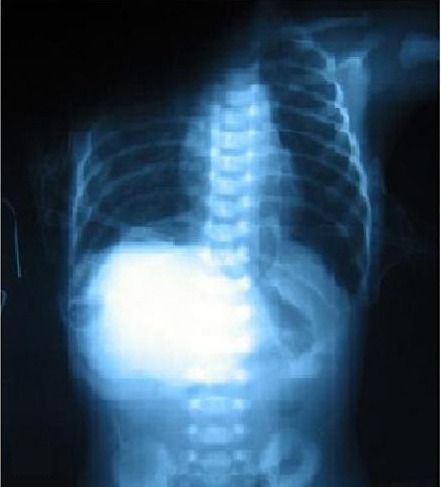
Figure 3: Postoperative chest radiograph.

The contents were reduced, sac excised and the defect closed with polypropylene sutures on both sides. Intercostal tubes were placed on both sides. After reducing liver and other contents, primary closure of the abdominal wall was not possible. Hence a silo was created using a polypropylene mesh sandwiched between two layers of Ioban TM (3M, Minnesota, USA). Postoperatively the baby was electively ventilated at rates of 50 per minute and FiO2 of 80%. The silo was further reduced by 1.5 cm after 24 hours. The patient, however, deteriorated and developed severe pulmonary hypertension. Although the lung showed reasonable expansion on a post operative chest radiograph (Fig. 1B), the child went into refractory shock and died 30 hours after surgery.

## DISCUSSION

CDH occurring in the neonatal period is mostly of the Bochdalek type. The defect is usually unilateral and involves the left diaphragm in 75% of cases [5]. On the contrary, bilateral CDH is rare and usually fatal. 

Antenatal diagnosis of CDH is relatively easy and the sonographic indicators of unilateral CDH are mediastinal shift, presence of intestine in thorax, small abdominal circumference and polyhydramnios [6]. Mediastinal shift is often the first abnormality observed. In our case the diagnosis was missed. Song and workers [7] in 2001 reported antenatal detection of bilateral CDH in a fetus and concluded that bilateral diaphragmatic hernia should be suspected when the degree of mediastinal shift is less than that anticipated for a unilateral hernia in presence of other features of CDH.

The first survivor of bilateral CDH in UK was reported in 1990 [8]. In 1987 Furuta et al [2] presented the 11th reported case of bilateral CDH. The patient expired in the postoperative period after repair of right CDH while the left side CDH was identified at autopsy. In our case there was no preoperative suggestion of left sided hernia. We discovered the contralateral CDH only at the time of operation when difficulty in reduction of liver was encountered. We favour an abdominal approach for all diaphragmatic hernias including the right CDH as the liver can be best handled and mobilized through the abdominal approach. In our case part of the right lobe had herniated through a defect in the diaphragm. The edge of the diaphragm caused an indentation in the liver. This produced an hour glass configuration with a large portion of liver inside thorax, a narrow “neck” and another large portion in the abdomen. Simply pushing it from above from a thoracic approach would have been quite difficult. Further the left sided pathology would have been completely missed. Also a silo can only be used with abdominal approach. 

Kufeji and Crabbe in 1999 reported familial inher-itance of bilateral CDH in two consecutive siblings with a similar kind of bilateral CDH [9]. Both the sib-lings in their report could not be diagnosed antenatally. The outcome of majority of these cases is poor. In 2003 Neville et al retrospectively reviewed cases of CDH treated in 83 different hospitals in USA. They found that in bilateral CDH mortality rate was 65% compared with 33% of patients with unilat-eral CDH [4]. In contrast, anecdotal reports of patients with good outcome have also been published in literature. Zaupa et al in 2007 reported a case of bilateral CDH with gastroschisis with a favorable out-come [10]. They hypothesized that a low intrathoracic pressure due to gastroschisis may have been responsible for good lung development and a good patient outcome. In our case although the preoperative stabilization was good, there was severe pulmonary hypertension that led to the mortal outcome. In conclusion, our case highlights the diagnostic difficulty that can occur in these rare cases.

## Footnotes

**Source of Support:** Nil

**Conflict of Interest:** None declared
